# Welfare Health and Productivity in Commercial Pig Herds

**DOI:** 10.3390/ani11041176

**Published:** 2021-04-20

**Authors:** Przemysław Racewicz, Agnieszka Ludwiczak, Ewa Skrzypczak, Joanna Składanowska-Baryza, Hanna Biesiada, Tomasz Nowak, Sebastian Nowaczewski, Maciej Zaborowicz, Marek Stanisz, Piotr Ślósarz

**Affiliations:** 1 Laboratory of Veterinary Public Health Protection, Department of Animal Breeding and Product Quality Assessment, Poznan University of Life Sciences, Słoneczna 1, 62-002 Suchy Las, Poland; hanna.biesiada@up.poznan.pl; 2 Department of Animal Breeding and Product Quality Assessment, Poznan University of Life Sciences, Słoneczna 1, 62-002 Suchy Las, Poland; agnieszka.ludwiczak@up.poznan.pl (A.L.); ewa.skrzypczak@up.poznan.pl (E.S.); joanna.skladanowska-baryza@up.poznan.pl (J.S.-B.); sebastian.nowaczewski@up.poznan.pl (S.N.); marek.stanisz@up.poznan.pl (M.S.); piotr.slosarz@up.poznan.pl (P.Ś.); 3 Department of Genetics and Animal Breeding, Animal Reproduction Laboratory, Poznan University of Life Sciences, 60-637 Poznan, Poland; tomasz.nowak@up.poznan.pl; 4 Institute of Biosystems Engineering, Poznan University of Life Sciences, 60-637 Poznan, Poland; maciej.zaborowicz@up.poznan.pl

**Keywords:** pigs, welfare, health, herd management, monitoring technologies

## Abstract

**Simple Summary:**

The continuous development of innovative technologies and the large-scale implementation of these solutions on farms are dynamically influencing the so-called precision livestock farming—PLF. Pig producers striving to increase the profitability of production, food safety, and food itself are increasingly willing to invest in rationalisation systems that raise the technological standards of pig breeding. The use of modern systems in livestock management and animal welfare is based on the use of non-invasive monitoring devices such as cameras, microphones, or detectors and information technology-based data archiving and management systems that support farmers/breeders in the daily running of the farm. Precision farming technologies, which are beneficial for animal welfare as well as for the profit of the livestock producer, help to solve the problems of large-scale animal production and satisfy the expectations of food regulators and consumers themselves. The aim of the paper was to gather contemporary knowledge on innovative technologies applied on pig farms. The paper presents and compares methods of controlling herd behavioural parameters with the use of various monitoring systems and their purpose. The paper also includes a review of potential limitations that may occur in the daily use of the above-mentioned devices. The review presents results on the effectiveness of their use.

**Abstract:**

In recent years, there have been very dynamic changes in both pork production and pig breeding technology around the world. The general trend of increasing the efficiency of pig production, with reduced employment, requires optimisation and a comprehensive approach to herd management. One of the most important elements on the way to achieving this goal is to maintain animal welfare and health. The health of the pigs on the farm is also a key aspect in production economics. The need to maintain a high health status of pig herds by eliminating the frequency of different disease units and reducing the need for antimicrobial substances is part of a broadly understood high potential herd management strategy. Thanks to the use of sensors (cameras, microphones, accelerometers, or radio-frequency identification transponders), the images, sounds, movements, and vital signs of animals are combined through algorithms and analysed for non-invasive monitoring of animals, which allows for early detection of diseases, improves their welfare, and increases the productivity of breeding. Automated, innovative early warning systems based on continuous monitoring of specific physiological (e.g., body temperature) and behavioural parameters can provide an alternative to direct diagnosis and visual assessment by the veterinarian or the herd keeper.

## 1. Introduction

In recent years, the world has seen rapid changes in the dynamics and efficiency of pig production. The general trend of increasing production, with reduced employment, requires optimisation and a comprehensive approach to herd management [1]. Modern pig production should therefore be based not only on a modern infrastructure and a precisely designed feeding program, but also on the use of modern technologies for monitoring health and welfare of the entire herd [2,3,4].

Herd health programs for swine include biosecurity, routine health control, and other preventive procedures allowing maintenance of a high health status of pig herds [5]. The health of pigs on the farm directly translates into the economics of production. Diseases lead to higher morbidity and mortality in different age groups and higher veterinary costs for the purchase of medicines and vaccines and more frequent veterinary visits [6,7]. Herds with low health status are also characterised by low productivity, reduced growth, and higher feed consumption. As a consequence, the fattening period is extended and the production efficiency is decreased [8].

Productive performance of pigs is a reliable indicator of the efficiency of production under different housing conditions [9,10]. The criteria for assessing animal welfare cover even more characteristics, including indicators of health and ethological parameters [11]. In practice, it is difficult to identify one basic and easy-to-use measure, which demonstrates the imperfections of each indicator and, on the other hand, the complexity of the concept of welfare [12].

In recent years, the market of equipment and systems for continuous, automatic health and behaviour monitoring in pig herds has been enriched by innovative technologies. Modern pig production systems based on intelligent technologies allow for planned, efficient, and thus more cost-effective production [5]. Considering the used methodology and the scope of application, three categories can be distinguished among the available devices. The first category devices are only aimed at detecting specific animal behaviour by means of special sensors. An example of such a solution is the system automatically measuring the frequency of pig visits to the feeder and the time taken to feed by means of radio frequency identification technology—RFID [13,14]. Another example is the use of real-time video visualisation using conventional (2D) monochromatic or colour cameras or 3D cameras to depict activity level, area occupancy, aggression, gait scores, resource use, and posture [15,16,17,18]. The second category devices allow for detection and recording of specific behaviours, such as drinking [19], feeding [20], or spatial distribution [21], which are further processed into numeric data and presented, e.g., in the form of a graph on a mobile phone monitor. This type of device allows for identification of changes in animal behaviour but requires farm workers to interpret the data. The last category involves intelligent production systems, automatically analysing the recorded changes in the physiological and behavioural parameters. These systems are based on optimal settings of farm environment, have the ability to extract deviations from theses settings, and automatically make decisions to adapt the production environment to optimal production conditions [22,23]. The goal of this paper was to gather knowledge on novel technologies applied on pig farms in order to promote their health, productivity, and welfare. The article should be in the interest of pig farmers, pork retailers, and researchers working in the field of meat science.

## 2. Challenges of Pig Farming

There were about 677.6 million pigs worldwide as of January 2020 [24]. Pork is the second-most consumed meat in the world, with the consumption reaching 23.0 kg/capita [25]. Population growth increases demand for meat. The statistics on the projected pork consumption indicate a global increase, by about 17%, predicted for the period from 2021 to 2029 [26].

Modern pig production is characterised by intensification and specialisation of production. These two factors lead to an increase in animal productivity and thus contribute to higher economic efficiency of production. On the other hand, they cause serious ecological problems as well as problems related to animal welfare, herd health, and food safety [27]. A high level of welfare is a guarantee of good health of the animal as well as the elimination of antibiotics or other drugs [28].

A series of programs were launched in European Union in order to evaluate and improve the welfare of farmed animals, with the Welfare Quality^®^ program resulting in the development of welfare protocols for the animal species under large-scale production [29,30,31,32]. Welfare Quality^®^ protocols contain major welfare principles targeted at the needs of animals under intensive production and based on the Five Freedoms of animal welfare [33]. The protocols are designed in a manner that allows for their species-specific adaptation. According to the available research results, the protocol dedicated for pigs is a useful and reliable tool for identification of farms keeping pigs at poor level of welfare [34].

In order to prevent economic losses due to diseases occurring in the herd, pig farmers should acquire at least basic skills to diagnose and deal with the appearance of the disease unit in their shed. [35]

Changes in animal behaviour preceding or accompanying subclinical and clinical signs may be of significant diagnostic value. They are often referred to as sickness behaviour, including changes in eating habits, social behaviour, mobility, and posture. By definition, subclinical disease is latent, and thus direct monitoring based on staff observation is ineffective. This is due to the fact that it is time-consuming, inaccurate, and impractical in terms of work organisation [22,36].

Automated early warning systems, based on continuous monitoring of specific physiological (e.g., body temperature) and behavioural parameters, can provide an alternative to direct observation of animals [23,37]. A good example are methods based on artificial intelligence. These methods employ computer tools able to track animal behaviour [38,39,40,41,42] and distinguish individuals from each other [43].

Today, commercial pigs are exposed to a great number of stress factors, including stocking densities, high concentration of animals in a limited area, limited possibilities of movement and motivated behaviour expression, and frequent regrouping of animals. The microclimate in pig buildings also has a huge impact on pig welfare and production results. It affects animal health, reproduction parameters, and feed intake [37]. Harmful consequences of stress depend on the sensitivity of the animals to stressors as well as their severity and duration of action. Among farm animals, pigs are characterised as having the lowest tolerance to high environmental temperatures. This is due, among other things, to low adaptability of the thermoregulatory medium in the brain, low number of sweat glands, presence of the subcutaneous fat layer, and intensive metabolism. Exceeding the body’s ability to adapt to high temperatures (hyperthermia) is a threat to health and life [44,45]. Hyperthermia in pigs leads to poor condition of the animals, decreased daily gains, and longer fattening period. Moreover, it negatively affects the quality of pork resulting from the interaction between the muscle pH value and the temperature in the process of post-mortem denaturation of muscle proteins [46].

Among the consequences of stress, one can name reduced appetite, reproductive disorders, and reduced immunity. In the aftermath, the pig farmers observe lower daily gains, poor reproductive results, and the appearance of infectious diseases and high mortality [47].

## 3. Welfare Monitoring Systems for Pigs

The market of systems designed for permanent, automatic monitoring of farm animal welfare is constantly evolving. Automated innovative early warning systems (PLF—precision livestock farming), based on continuous monitoring of specific behavioural and physiological parameters, are an alternative to direct visual assessment by staff or veterinarian. Fast and accurate acquisition of real-time data on animal movement and feed intake frequency enables early detection of diseases and facilitates further management of the herd. Thanks to sensors (cameras, microphones, accelerometers, RFID sensors, and temperature sensors), behavioural patterns of animals are gathered and combined through algorithms. The data derived from PLF technologies can be used to derive warnings and trigger notifications and alarms [48]. With the development of the Internet of Things (IoT, i.e., the interconnection between computing devices via the Internet), decision making can be better informed by connecting PLF information with other data streams, and components of farm management can be automated or even controlled remotely [15,49,50]. This allows for the ability to detect problems early enough to prevent potential, negative effects on productive performance of animals [51]. There are many benefits of precision livestock production, including increased productivity and profitability, increased safety and quality of animal products, and improved animal welfare, as well as reduced environmental impact and combating climate change. The use of precision livestock production in animal nutrition has been shown to reduce feed costs by up to 25% [52,53]. In 2016, the total turnover in the precision agricultural technology market was estimated at USD 4.8 billion. Current forecasts put the market turnover in this area at USD 12.6 billion by 2025 [54]. The adoption of these technologies varies considerably. RFID and accelerometer technologies are well integrated, but other technologies still have to achieve a viable market share [48].

### 3.1. Vision-Based Systems

Among the adopted PLF methods, video monitoring seems to be the most commonly implemented. It provides non-invasive and efficient tools to be able to record not only the behaviour of a group of animals but the behaviour of each individual [35]. By means of image analysis, the results are converted to detailed data on animal distribution (location and proximity) [18,55] and activity (position and movement) [56,57]. Imaging is also used in pigs to measure body weight [58,59,60] and to detect lameness [61], aggressive behaviour [62], and heat [63].

Over recent decades, two-dimensional (2D) monochrome and colour have been widely used in computer vision due to its low cost and high efficiency. Many researchers have proposed different systems to extract livestock characteristics, such as body size or body condition, on the basis of 2D images [64,65]. For example 2D image analysis allows for monitoring and estimation of pig growth rates to an accuracy of 1 kg [58]. In turn, the number of cameras (video sets) intended for animal observation depends on the monitored area and the height at which the camera is placed. The quality of monitoring is also influenced by the number of animals per square meter [22]. As many studies show, simple dome cameras are sufficient to monitor the behaviour of the inmates [41,65,66,67]. For example, it can be a CCTV camera with IP67 waterproof rating and 2MP (1080) resolution, with f/2.8/F1.6 minimum aperture and with built-in infrared heater [67]. Monochrome cameras typically have greater light sensitivity and are thus more ideal for recording under lower light conditions than colour cameras [15].

In the work of Chen et al. [65], a behaviour identification and monitoring study was conducted on eight pigs in pens of approximately 4 square meters, with the camera positioned at a height of 2.4 m. This study used the neural modelling technique using deep learning algorithms. It should be noted that the authors used a more difficult modelling technique in relation to convolutional networks and searched for their own indexes to describe the image. They obtained 98.5% accuracy in terms of behaviour identification through connection of a relatively simple camera with neural modelling technology. The results of research conducted by Chen et al. [65] allow for high efficiency of algorithms used in systems for monitoring animal behaviour [65]. Reikert et al. [67] applied the deep learning system in combination with 2D cameras in order to detect the position and posture of pigs. The authors obtained slightly lower precision results compared to the previously described study (87.4 and 80.2%), but the area of pens and the stocking of animals were much larger [67].

Despite continuous development, 2D imaging technology still has some limitations. It requires appropriate ambient lighting; provides only a flat projection of the animal [68]; is influenced by distance, wavelength, and applied filters [69]; and also requires a contrasting background, e.g., a bright pig against a dark pen wall [15]. Additionally, data extraction from images taken in various environmental conditions leads to inaccurate operation of computer tools for image processing and analysis [70,71].

Three-dimensional (3D) (RGBD) cameras equipped with high-resolution lenses, infrared sensors, or depth sensors with time of flight (ToF) technology give greater possibilities compared to cheaper two-dimensional (2D) ones [35]. ToF technology sends a pulse of infrared light from LED several times per second and records the delay between the pulse and its return. The 3D cameras can operate regardless of the visual light environment, including in total darkness; are unaffected by changing light conditions including changes in contrast and shadow; and are less prone to errors due to occlusion [15,17]. Three-dimensional technology opens the possibility to reconstruct the geometry of animals’ bodies and to link abnormal morphological changes to behavioural changes [7,72]. Cameras equipped with ToF technology (kinect cameras) are extremely useful in precision animal husbandry due to their relatively low cost, ability to handle large databases, low power requirements, and ability to adapt to changing light and background conditions [35,73]. However, these types of equipment have a limited distance range (i.e., up to 4.5 m), and the accuracy of the depth data measured by such devices decreases squarely with increasing distance [74].

Despite these limitations, the ability of kinect cameras to detect the movement of individual animals is satisfactory. This was proven in the study of Kim et al. [74], which shows that one Kinect set installed at a height of 3.8 m is sufficient for accurate (94.47% sensitivity) monitoring of an area measuring 2.4 by 2.7 m.

Although video-based recognition of pig behaviour has made significant progress, there are still some unsolved problems [71]. Vision data may require considerable processing and there have been studies on the trade-off between the video image quality and computational processing requirements [75]. Software challenges include detecting individual pigs on the basis of selected features by means of feature selection algorithms [76]. In addition, cameras are susceptible to dust and damage from ammonia, being part of the pig farms’ environment, although this can potentially be negated through ingress protection enclosures and maintenance [77].

To ensure accurate and continuous monitoring of individual animals on a modern livestock farm, farmers today need reliable and inexpensive technology [3]. Such systems already exist in cattle breeding and include GEA CowView system, or Lely Qwes. For pigs, the RO-MAIN Smart Cam and eYeNamicTM system is currently the best-known system used to identify and track the pattern of activity in a group of pigs during the growing period. Other newer solutions are still in the realm of research [48].

### 3.2. Sound-Based Systems

Real-time monitoring can be carried out not only by camera and image analysis but also by microphone and sound analysis [78]. Audio recordings combined with voice analysis and machine learning algorithms are used to detect heat stress and conditions of illness or suffering in animals [5]. Respiratory diseases and/or discomfort associated with poor air quality can cause changes in vocal characteristics and some acoustic signs, such as coughing and sneezing [79]. Monitoring coughing is particularly useful, as it can be easily distinguished from other sounds [80]. Specialised microphones or groups of microphones (microphone arrays) give the ability to distinguish infectious cough from coughs caused by accumulated ammonia or dust, and allow for automated sound source location [81]. Currently available sound analysis systems are so accurate that they can detect and locate respiratory disease outbreaks between individual pens [82]. Several studies have taken up the subject of animal coughing sounds analysis, under laboratory and farm conditions [82,83,84]. The first study on cough detection in pigs was conducted by Van Hirtum et al. [85] and was followed by additional research on refining algorithms for pigs’ cough detection [82,84]. In the study of Ferrari et al. [86], the authors used cough-sound analysis to identify respiratory tract infections in pigs. They found significant differences in several major acoustic parameters, including peak frequency, duration, and time occurring between consecutive coughs in healthy and infected pigs. In turn Exadaktylos et al. [84] proposed a method to identify sick pigs in real time by analysing the sound of coughing, with a recognition accuracy of 85%. Research results on the application of cough algorithms allowed for the development of a commercial tool, the respiratory distress monitor, able to detect infected pigs 2-12 days before the farmer or veterinarian [87]. Van Hirtum and Berckmans [88] suggested that cough sound recognition could be used as a biomarker of air pollution. This thesis was confirmed in a recent study of Wang et al. [79]. Unfortunately sound detection and analysis on pig farms is impeded by the noisy environment of the farm [80].

### 3.3. Temperature-Based Systems

Temperature meters typically use thermometers embedded in a data logger or a sensor installed in the ear tag or subcutaneous transponder [89,90,91]. However, as Hartinger et al. [91] reported, this method is characterised by a high degree of variability, which makes it moderately reliable. It has been reported [92] that subcutaneous implanted transponders show a temperature around 1 °C lower compared to rectal measures. Explanation of this discrepancy lies in the position of the transponder, the amount of adipose tissue in the region of measurement, behavioural factors, environmental changes, heat radiation, or blood perfusion in the connective tissue in the implant area. Lohse et al. [92] have shown in their studies that transponders introduced into skeletal muscles show a better correlation with rectal temperature than transponders introduced subcutaneously. An alternative to invasive body temperature measurement with a transponder is to measure the temperature distribution on the body surface by using thermal imaging. Thermography, also known as thermovision, is a method of remote and non-contact assessment of body surface temperature distribution. This technique allows for visualisation of infrared radiation, and thus can obtain information about physiological and pathological processes taking place in the body of humans and animals [93]. The application of thermovision is absolutely non-invasive and has no risk of spreading infections [94,95]. In the diagnosis of farm animals, thermovision is used to investigate injuries and inflammation of the locomotor system, detect infectious diseases, diagnose heat and pregnancy, and monitor welfare and stress levels [93]. Modern thermovision methods make it possible to determine temperature changes both in terms of values and spatial distribution, both in static and dynamic terms. Thermal imaging cameras can produce high resolution images with a temperature accuracy of up to 0.08 °C [96]. Temperature readings depend on the animal’s temperature, environmental conditions, and thermoregulation of the peripheral circulatory system. At higher ambient temperatures, thermoregulation results in increased blood flow to the skin tissue, causing an increase of surface temperature [97]. In adult pigs, the temperature measured on the body surface is lower compared to younger animals due to the insulating effect of subcutaneous fat [97]. Skin surfaces behind the ears or near the sternum are hairless and lacking in fat insulation, and therefore better reflect adult body temperature [94].

### 3.4. Activity-Based Systems

Accelerometers are among the most promising technologies for monitoring livestock behaviour [35]. These instruments are primarily used to measure linear or angular acceleration, and allow for very accurate monitoring and analysis of animal activity: posture and walking patterns, the length of time it spends standing up, delays in lifting, or even antepartum activity in pens, making it possible to detect the onset of labour in sows [98]. Triaxial accelerometers allow for the possibility of collecting three-dimensional information and measure the earth’s force by determining the angle of a device (e.g., wireless acceleration sensor nodes placed on the back to record the three-axis movement of pigs) and by measuring the acceleration forces [35]. Several studies have described automatic detection by accelerometers of standing and walking behaviour in pigs [99,100,101]. Studies of accelerometer readings installed on ear tags have shown that although the ear is virtually independent of the animal’s locomotor system, the range of data provided by the device is sufficient to reliably detect early lameness in pigs [102]. Other research indicate that a combination of data from accelerators with data from body temperature sensors allows for automatic detection of infections 1–3 days before using specific diagnostic methods [23]. Thus far, high accuracies have been found for movement and resting behaviours in cows and pigs, while the development of algorithms for analysing feeding and drinking behaviours in pigs is far behind these developed for cattle [103].

Disease, welfare, and productivity problems can have an impact on the feeding patterns of pigs, and may lead to a reduced feeding time or longer intervals between feed intakes [104,105]. RFID at feeding and drinking areas has been used to measure occurrence and duration feeding and drinking behaviour of individual pigs’ [20,106,107]. An RFID system requires an RFID transponder (ear tag) and an RFID antenna or receiver (located at the feeder or drinker) [22]. The device is implanted primarily in the ear tags and stores information such as the animal’s unique identification number and farm identification number. These data can be used immediately to identify individuals or can be stored and analysed later [35]. Low-frequency RFID is used, for example, in electronic feeders, and makes it possible to dose individually adjusted feed rations [108]. At the same time, data from RFID readers are also used to analyse the frequency of visits to the feeders and the time taken to feed, which allows for early detection of behavioural signs of health problems [107]. Nevertheless, low frequency RFID has two main disadvantages: low reading range (<1 m) and the impossibility to identify more than one animal at a time within the reader’s range [104,109]. There is research on the application of high-frequency UHF readers to track multiple animals simultaneously and at longer ranges (3–10 m) [110,111]. Such systems often include anti-collision algorithms to avoid data loss when multiple tags are within the reading range [112,113]. Thanks to its high sensitivity (88.58%) and specificity (98.34%), the HF RFID system performs well in recording feeding visits of pigs [105]. An example of a commercial system used in pig farming, based on UHF-RFID technology, is the SLIDE^®^ system (Simplum Gliwice, Poland). One of the key elements of this system, which distinguishes it from other similar solutions, is the long reading range of approximately 4–5 m, allowing for the automation of the data acquisition process. The system allows not only for monitoring of animals, but also offers the possibility of a very detailed analysis of the individual indicators of each tagged pig, taking into account the factors influencing them and the relationships between individuals [114].

RFID solutions are eagerly used in various sectors, e.g., in factories and warehouses. However, it is important to be aware that the conditions in pig stables, which are mostly based on concrete and reinforced structures, may disrupt the transmission of waves and data. Other disadvantages of RFID-based technology include frequent loss or failure of tags, pain and stress for the animal during tagging, and the need to remove the tag prior to slaughter [35]. 

An idea worthy of attention is the use of beacons—microcontrollers equipped with BLE (Bluetooth Low Energy) transmitters in pig farming as devices used to identify behaviour and physiological condition. Studies on these systems have been successfully carried on cattle, although the difference in the daily behaviour of cows and pigs is important [115]. Table 1 summarises the advantages and disadvantages of equipment used in precision pig farming.

## 4. Automatic Health and Welfare Monitoring Systems for Pigs—Farmer and Consumer Perspective

Automatic systems for health problem detection in pigs are practical from the scientific point of view and are undoubtedly a common topic in research on detection of health problems on commercial pig farms. The automatic health and behaviour measurement seem to perfectly fit management of big stables; it allows for recording and storing of valuable data, and thus carrying out continuous observations on large numbers of pigs. However, the major problem remains unsolved: How do we convince pig farmers to adopt novel solutions? Are these solutions economically profitable? The economic aspect of health monitoring systems for pigs is still undefined, as no research has been made to compare the outputs and the costs of all inputs used. Moreover, the popularity and possibility of implementation of automatic systems for pig health monitoring is affected by a group of additional factors: effectiveness and reliability of measures, the awareness and technical knowledge of pig farmers, and the housing system and herd size used [116].

Recent years have seen a series of major leaps forward in the technologies and methods of automated animal observation and monitoring, notably under the generic terminology of PLF whose general aim is to increase the efficiency of livestock farming systems while reducing the workload [2].

Commercial pig farms are an aggregation of technical solutions that allow for increased production while limiting the labour. In times of sustainable agriculture and high welfare farming, the commercial pig farms have limited opportunities to follow these trends and to compete with the good reputation of organic pig farms. The modern pig production should meet the public requirements of animal welfare, and the use of automatic systems for welfare monitoring might be a chance of fulfilling these requirements. PLF technologies have the potential to monitor animal health and behaviour in ways that go beyond those of conventional welfare monitoring and observation. PLF allows for the establishment of welfare indicators that are not dependent solely upon periodic human observation and measurement [2]. In addition, PLF provides the opportunity to observe animal behaviour without interference.

Problems that need to be resolved in the near future include inter alia, technical, and scientific issues related to the definition of welfare. In addition, it is necessary to establish effective welfare indicators, improve the reliability of data collected using new observation technologies, develop welfare automation technology dedicated to extensive farming, regulate matters related to the ownership of data generated by PLF technologies in order to effectively manage these data, and further the possibility of exchanging such information between participants in the food chain. To date, however, this potential is both underdeveloped and under-studied [48].

Balzani and Hanlon [117] underline that animal welfare links a variety of perspectives: animal science, veterinary science, public opinion, and the perspective of the farmer, which is often disregarded. Therefore, implementation of automatic welfare monitoring systems should be preceded by research that relate science with farm practice and social reaction. The practical aspects such as economic profitability and consumer feedback should be carefully analysed as this kind of research is lacking. With growing concern for animal welfare, the pressure on the implementation of PLF technology in pig farms will also increase, both from food chain operators and consumers [48,118]. The question is how will consumers feel about the use of novel technologies to assist the farmer with the monitoring of the welfare of pigs. Are they willing to pay higher price for pork produced with this extra supervision? How much educational input is required to create social attitudes that favour novel technologies? The automatic health and welfare monitoring systems in pig stables should be promoted as one of the paths in sustainable animal production, and one of systems that allow for production at high welfare standards. A pork production chain that employs use of systems supporting health and welfare of pigs should be traceable for consumers and promoted by the authorities of each country.

The farmer’s attitude to pig welfare monitoring systems is even more important than the social opinion. Because the EU directive (Council Directive 98/58/EC) defining the rules of pig farming charges farmers and stock-people with the responsibility to inspect animals at regular intervals (usually, at least once a day) to verify their wellbeing, farmers’ knowledge of the biological and behavioural needs of pigs is key to bringing about changes that promote welfare automation in the pig industry [119,120]. If the farmer is aware that the stable requires changes to produce pigs at high welfare standards, the decision on implementation of novel technological solutions will depend on their economic profitability.

## 5. Conclusions

This review concluded that automatic health monitoring systems should be widely implemented into the pig industry in order to increase the effectiveness of healthy pig production. The monitoring systems are developing together with the knowledge on effective animal production, requirements considering the level of welfare, and the developments in available technologies. Implementation of novel technologies for health monitoring may be an answer to the demands of society and animal welfare organisations. The research in the field of swine industry deliver a number of practical solutions. The solutions that are already commonly used are automated weight measurement, electronic identification of pigs, automated measurement of feed and water intake, accelerometers (in ear tags) measuring activity, and systems to monitor and manage the microclimate (humidity, temperature, ventilation). The automatic welfare monitoring systems give much more data on the pig herd and are much more developed that these commonly used technologies. However, without proving their economic profitability and defining the reliable possibilities of application, automatic health/welfare monitoring systems will not gain popularity. Research is lacking in this field. Though the social pressure may be the “drive motor” of changes in animal production, the economic aspects of pig heath monitoring systems will decide on the scale of their implementation. Acquisition of data defining the health on pigs in real-time is the key to early disease recognition and disease prevention. The expectations considering the monitoring systems gradually increase, and with time we can observe new technologies that allow us to trace individuals and monitor pigs without stressing the animals.

Summing up the issues discussed in this review, one last thing is still lacking—a system that will allow us to link the health and welfare measures of an individual pig with the data on the quality attributes of obtained pork. Only the careful analysis of this relation would allow for a reliable assessment of the role of pigs’ health and welfare in the economical effectiveness of the swine industry, aimed at the production of high-quality meat.

## Figures and Tables

**Table 1:** Advantages and disadvantages of equipment used in precision pig farming.

Equipment	Application	Advantages	Disadvantages
2D (RGB) cameras	Pig identification based on detection of colours in the image [67]. Automatically detecting pig locomotion [56]. Automatically detecting pig position and posture [21]. Monitoring the environment in a pig pen [21]. Analyse the group behaviour of pigs [21].	Non-invasive method [67]. Possibility of individual or group analysis [67]. Helps to analyse how often animals visit the feeder [48]. Helps determine time of animal feed intake [48].	Performance depends on lighting conditions [67]. Very similar appearances of pigs and varying statuses of the background [41]. Vulnerability to errors due to occlusion [15]. May require protective shielding against environmental factors [71]. Requires filtering to obtain useful information [70].
3D (RGBD) cameras	Estimation of pig body weights [60]. Identification of standing pigs [74]. Tail biting detection [67]. Automatically detecting pig locomotion [61].	Non-invasive method [67]. Possibility of individual or group analysis [67]. Ability to handle large datasets [35]. Ability to adapt to variable light and background conditions [74].	May require protective shielding [71]. Limited depth measurement range [67]. Vulnerability to errors due to occlusion [15].
Microphones	Detection of sickness and heat stress [2]. Cough detection [2]. Group behaviour monitoring [2].	Non-invasive method [2]. Monitoring of large groups of animals with a single sensor [2]. Indirect detection of air pollution [85]. Can be used indoor and outdoor [2].	Susceptibility to interference from environmental sounds [82]. Environmental factors may interfere with the functioning of the microphone [82].
Thermometers (implantable device)	Measurement of the body temperature [93]. Monitoring of physiological reactions [94].	Useful for detecting temperature variations [94].	Invasive method (transponders) [94]. Moderately reliable method [92].
Infrared thermal imaging (IR)	Remote temperature measurement [93]. Temperature monitoring of the whole herd and individual animals [93]. Examination of musculoskeletal injuries, detection of infectious diseases, diagnosis of oestrus and pregnancy, and monitoring of welfare and stress levels [93].	Non-invasive method [97]. Low light imaging capability [97]. Useful for the analysis of physiological processes [97].	Environmental factors may interfere with the measurement results [94]. High equipment cost [94].
Accelerometer	Pig movement detection and analysis [35]. Monitoring of pig activity: posture and walking patterns, the length of time it spends standing up [103].	Non-invasive method (collars) [102]. Useful for the analysis of movement [35]. Provides real-time readings [105].	Invasive method (ear tags) [102]. Requires external data analysis [103]. Sensors are fragile and prone to mechanical failure [103]. Requirement to remove the identifier before slaughter [35].
RFID transponders R	Pig identification [105]. Nutrition management [105].	Helps to analyse how often animals visit the feeder [104]. Helps determine time of animal feed intake [104]. Provides real-time readings [105].	Low range low frequency RFID reading [106]. Inability to identify more than one animal at a time within the range of the reader [106]. Requirement to remove the identifier before slaughter [35].

## Data Availability

No new data were created or analysed in this study. Data sharing is not applicable to this article.
